# A Case of *Brevibacillus brevis* Meningitis and Bacteremia

**DOI:** 10.1155/2020/5931235

**Published:** 2020-07-25

**Authors:** Parmvir Parmar, Milani Sivapragasam, Vicente Corrales-Medina

**Affiliations:** ^1^The Division of Infectious Diseases, Department of Medicine, University of Ottawa, Ottawa, ON, Canada; ^2^Ottawa Hospital Research Institute, Ottawa, ON, Canada; ^3^McGill University, Montreal, QC, Canada

## Abstract

*Brevibacillus* species are environmental organisms that are rarely implicated as human pathogens. We present the case of postsurgical *Brevibacillus brevis* bacterial meningitis and an associated bacteremia after debulking surgery for a newly diagnosed pilocytic astrocytoma in a 19-year-old woman. The patient experienced clinical cure with a 4-week course of vancomycin, but her postinfectious course was complicated by the development of a pseudomeningocele that required surgical repair. To our knowledge, this is the first described case of a central nervous system infection caused by *Brevibacillus brevis* in the literature.

## 1. Case Description

A 19-year-old student with a history of migraines and no other significant medical history underwent an outpatient MRI head due to worsening migraine symptoms. She was found to have a 2.5 cm tumor in the left cerebellum. She underwent a craniotomy, tumor debulking, and bone flap with a titanium mesh. A diagnosis of pilocytic astrocytoma was made from surgical pathology results on the postop day 4. The same day, her pathology results were reported, and she developed a fever of 38.1, nausea, vomiting, photophobia, 10/10 headache, and stiff neck. She was alert and oriented, hemodynamically stable, and found to have nuchal rigidity. Blood cultures were taken, and a lumbar puncture was performed. She was started on ceftazidime and vancomycin as empiric therapy for neurosurgical site infection. Her white blood cell count was 15.4 × 10^9^/L (3.5–10.5 × 10^9^/L). The CSF cell count was 13050 × 10^6^/L (0–5 × 10^6^/L), with 83% neutrophils, the CSF protein was 3.26 g/L (0.13–0.45 g/L), and the CSF glucose was 1.8 mmol/L (2.2–3.9 mmol/L). The gram stain from the CSF revealed many white cells and rare Gram-positive bacilli. The Gram stain from the blood culture also revealed Gram-positive bacilli in the aerobic bottle. The Gram-positive bacilli in blood and CSF were later speciated as *Brevibacillus brevis* by MALDI-TOF.

Once *Brevibacillus* was identified, the ceftazidime was stopped. Vancomycin was continued, and meropenem was added. She defervesed, and her meningismus resolved. Her white count also normalized.

Antibiotic susceptibilities from the *Brevibacillus brevis* isolate from her blood were performed at the Public Health Ontario Laboratories in Toronto, Ontario, and reported as susceptible to ciprofloxacin and vancomycin and intermediate to clindamycin. Meropenem was discontinued, and she was maintained on intravenous vancomycin. She was discharged to a rehabilitation center to participate in neurologic rehab.

On her 4^th^ week of vancomycin therapy, she developed a morbilliform rash that was thought to be due to vancomycin. Vancomycin was discontinued, and she was maintained off antimicrobial therapy. She was afebrile, and her meningismus had entirely resolved. She reported persistent headaches, which were less severe than her initial presentation. She had a repeat MRI that demonstrated the development of a pseudomeningocele for which she underwent an open repair of her dura.

## 2. Discussion

*Brevibacillus brevis* (formerly named *Bacillus brevis*) is an aerobic, spore-forming Gram-positive *bacillus* [1–3].*Brevibacillus* species are known to produce a range of antimicrobial peptides, and *Brevibacillus brevis* produces gramicidin [1, 2, 4]. *Brevibacillus brevis* is also exploited in agribusiness; spores are utilized in the fermentation of foods such as soybean paste in the Far East [5].

*Brevibacillae* are organisms that are found in the environment and soil [1, 2, 6]. *Brevibacillus* species are rarely pathogenic, but can cause severe infections in immunocompromised hosts, intravenous drug users, victims of burns and physical trauma, dialysis patients, and patients who have undergone recent orthopedic and neurosurgical procedures [5].

For the specific case of *Brevibacillus brevis*, there is a paucity of the literature describing infections by this organism in humans. We were able to find only two case reports of *Brevibacillus brevis* infection, one describing bacterial peritonitis in a patient with hepatocellular carcinoma and another involving bacterial tracheitis in a patient admitted to the intensive care unit [5, 7]. To our knowledge, this is the first case describing a central nervous system infection with *Brevibacillus brevis*.

Given the exceedingly few published clinical cases of *Brevibacillus brevis*, descriptions of successful treatment regimens are limited. As with other *bacillus* species, vancomycin is reported to be the drug of choice [4, 5]. Most *Bacillus* species are also susceptible to clindamycin, fluoroquinolones, and aminoglycosides; susceptibility to penicillins and cephalosporins is more variable [4–6].

With the previously described cases of *Brevibacillus brevis* infection, clinical and microbiologic cure was achieved with 5 days of intravenous vancomycin in the case of the patient with peritonitis and with 5 days of teicoplanin in the case of the tracheitis [5, 7].

With an increasing use of MALDI-TOF and molecular methods, the number of *Brevibacillus brevis* isolates from clinical samples will likely increase. We also postulate that there will be an increase in infections attributable to this organism as the proportion of patients on immunomodulatory medications rises.
